# The influence of prosthetic suspension on gait and cortical modulations is persons with a transfemoral amputation: socket-suspended versus bone-anchored prosthesis

**DOI:** 10.1186/s12984-024-01331-y

**Published:** 2024-03-07

**Authors:** Vera Kooiman, Joris van der Cruijsen, Ruud Leijendekkers, Nico Verdonschot, Teodoro Solis-Escalante, Vivian Weerdesteyn

**Affiliations:** 1 Orthopedic Research Laboratory, Radboud University Medical Center, P.O. Box 9101, 6500 HB Nijmegen, The Netherlands; 2 Department of Rehabilitation, Donders Institute for Brain, Cognition and Behaviour, Radboud University Medical Center, P.O. Box 9101, 6500 HB Nijmegen, The Netherlands; 3 Radboud Institute for Health Sciences, IQ Healthcare, Radboud University Medical Center, P.O. Box 9101, 6500 HB Nijmegen, The Netherlands; 4 Department of Biomechanical Engineering, Faculty of Engineering Technology, University of Twente, P.O. Box 217, 7500 AE Enschede, The Netherlands; 5 Sint Maartenskliniek, Research & Rehabilitation, P.O. Box 9011, 6500 GM Nijmegen, The Netherlands

**Keywords:** Prosthesis, Amputee, Osseointegration, Socket, Gait, Cortical, EEG, Stability

## Abstract

**Background:**

Persons with a transfemoral amputation (TFA) often experience difficulties in daily-life ambulation, including an asymmetrical and less stable gait pattern and a greater cognitive demand of walking. However, it remains unclear whether this is effected by the prosthetic suspension, as eliminating the non-rigid prosthetic connection may influence stability and cortical activity during walking. Spatiotemporal and stability-related gait parameters, as well as cortical activity during walking, were evaluated between highly active individuals (MFC-level K3-4) with a TFA and able-bodied (AB) persons, and between persons with a bone-anchored prosthesis (BAP) and those with a socket-suspended prosthesis (SSP).

**Methods:**

18 AB persons and 20 persons with a unilateral TFA (10 BAP-users, 10 SSP-users) walked on a treadmill at their preferred speed. Spatiotemporal and margin of stability parameters were extracted from three-dimensional movement recordings. In addition, 126-channel electroencephalogram (EEG) was recorded. Brain-related activity from several cortical areas was isolated using independent component analysis. Source-level data were divided into gait cycles and subjected to time–frequency analysis to determine gait-cycle dependent modulations of cortical activity.

**Results:**

Persons with TFA walked with smaller and wider steps and with greater variability in mediolateral foot placement than AB subjects; no significant differences were found between BAP- and SSP-users. The EEG analysis yielded four cortical clusters in frontal, central (both hemispheres), and parietal areas. No statistically significant between-group differences were found in the mean power over the entire gait cycle. The event-related spectral perturbation maps revealed differences in power modulations (theta, alpha, and beta bands) between TFA and AB groups, and between BAP- and SSP-users, with largest differences observed around heel strike of either leg.

**Conclusions:**

The anticipated differences in gait parameters in persons with TFA were confirmed, however no significant effect of the fixed suspension of a BAP was found. The preliminary EEG findings may indicate more active monitoring and control of stability in persons with TFA, which appeared to be timed differently in SSP than in BAP-users. Future studies may focus on walking tasks that challenge stability to further investigate differences related to prosthetic suspension.

## Background

Persons using a transfemoral prosthesis encounter various challenges during daily-life ambulation, partly due to reduced sensory feedback, loss of muscle function, and therefore inability to actively control the joints in the prosthetic leg. Previous studies have found that persons with a transfemoral prosthesis walk slower, with shorter, wider and more asymmetric steps, longer step duration and larger toe clearance compared to able-bodied persons [1, 2]. These spatiotemporal adaptations point to the use of compensation strategies by persons with a transfemoral amputation (TFA) to maintain stability during gait [2–4]. Yet, despite such compensatory strategies, gait stability in people with TFA is reduced compared to able-bodied subjects [1, 3].

In addition, the prosthetic suspension may be another factor impacting gait stability in people with TFA. A socket is the conventional prosthetic suspension of a transfemoral prosthesis. Despite advances in material (e.g. liner) and fitting procedures, it is impossible to entirely prevent the residual limb from moving inside the socket during the stance phase due to inherent deformation of the soft tissue of the residual limb. This non-rigid connection between the body and the prosthesis may impact the gait pattern as well as stability during gait of prosthetic users. In recent years, a bone-anchored prosthesis (BAP) has been introduced as a new type of prosthetic suspension to resolve socket-related problems like pressure pain and wounds [5–8]. The fixed suspension between the residual limb and the prosthesis eliminates any potential movements in the socket. Whether this advantage of a BAP also benefits gait stability remains unclear.

Walking with a transfemoral prothesis leads to compensatory strategies and reduced gait stability, which may contribute to an increased cognitive demand for walking in persons with a TFA. Research has shown that the addition of a secondary cognitive task exacerbates gait asymmetry in persons with a socket-suspended prosthesis (SSP) [9], which indicates a cognitive contribution to walking. The addition of a secondary cognitive task has also been found to affect walking and standing balance to a greater degree in persons using a SSP compared to able-bodied persons [9, 10]. A study using fNIRS during walking reported increased cortical brain activity in the frontal and motor cortex in persons walking with a transfemoral prosthesis [11], suggesting that walking with a transfemoral prosthesis required additional motor planning and motor control. Yet, it remains unclear how this cortical activity may relate to specific gait-cycle events and which can be addressed by evaluating gait-cycle dependent cortical activations using mobile EEG. Evaluating cortical dynamics around heel strike is a topic of interest, as foot placement (i.e. timing and position relative to the moving centre of mass) plays a key role in controlling gait stability [12]. Previous research has shown that theta, alpha and beta modulations in the frontal central and parietal cortex are related to performance monitoring, motor planning and motor control, which plays an essential role in controlling gait and gait stability [13–22]. In addition, evaluating gait cycle-dependent modulation in cortical activity could provide insight into differences between prosthetic suspensions during gait in people with a TFA.

The first objective of the current study was to determine whether spatiotemporal and stability measures of gait differed between persons with a transfemoral amputation (TFA) and able-bodied persons (AB) in general; and, more specifically between persons using a socket-suspended (SSP) and those using a bone-anchored prosthesis (BAP). The secondary objective was to explore differences in cortical dynamics during gait between the aforementioned groups. Based on previous research [22, 23] on cortical activity related to gait stability, the current study focused on frontal and central alpha/beta modulations (i.e. related to motor planning and motor control) and frontal, central, and parietal theta modulations (i.e. related to performance monitoring).

## Methods

### Participants

For this cross-sectional study, 18 AB and 20 persons with a TFA were included. For the TFA group, we purposely recruited 10 persons using a SSP and 10 persons using BAP. All participants were highly active individuals (MFC-level K3-4). To be included, the persons with TFA should have used a BAP or SSP for at least 2 years and should not experience any prosthesis-related problems at the time of the measurement. Persons with neurological, vascular, or pulmonary diseases, or using medication affecting balance or gait were excluded from this study. Written informed consent was provided by all participants before participating in the study. The study procedures complied with the guidelines defined in the Declaration of Helsinki and were approved by the ethical committee CMO Oost Nederland (2018–4919).

### Study procedures and data recording

**Fig. 1:**
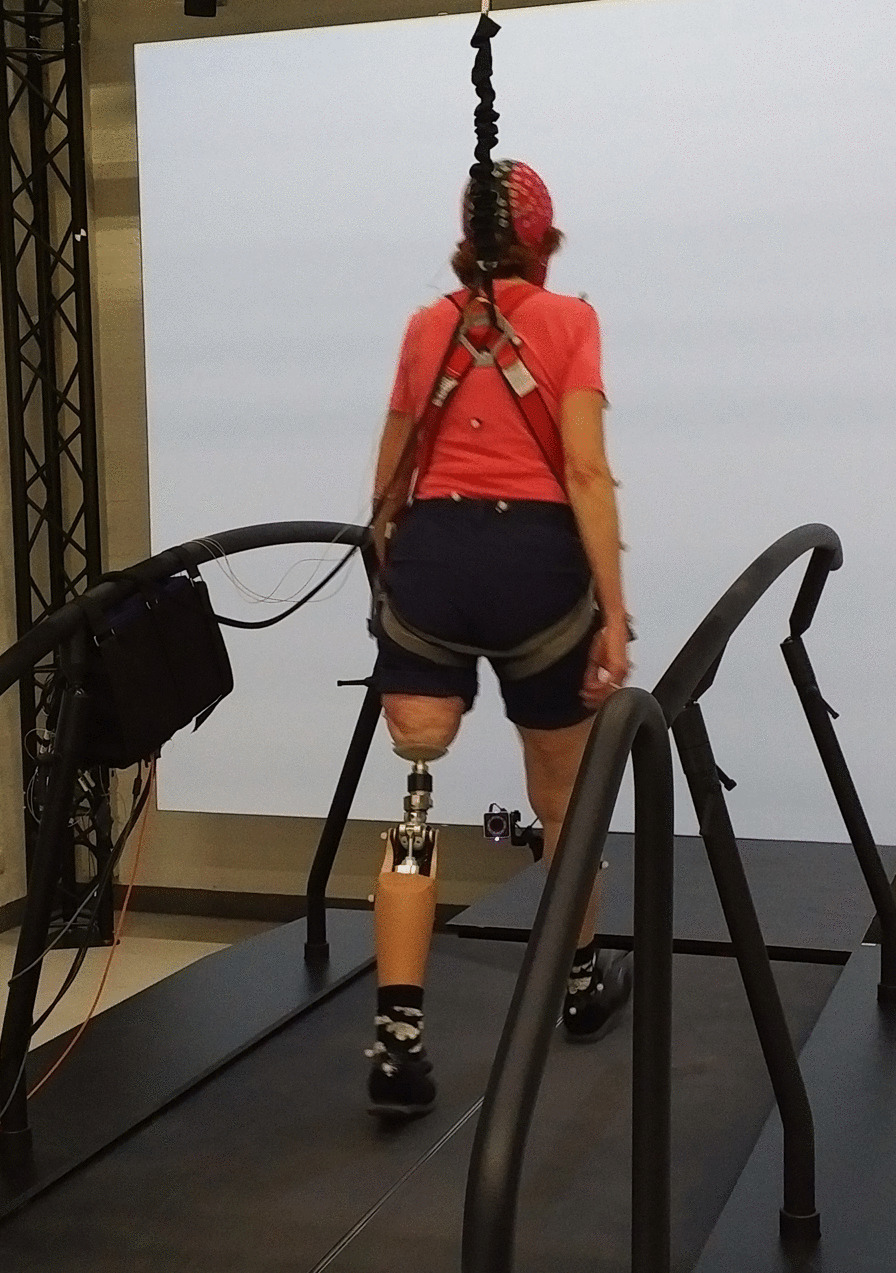
The experimental setup during the measurements

Participants were asked to stand still for 2 min to record brain activity during quiet stance. Subsequently, participants were familiarised with treadmill walking and the preferred walking speed was determined. During data recording, participants walked for 200 strides on an instrumented treadmill (M-Gait, Motek Medical) at their preferred walking speed (Fig. 1). Participants were asked not to talk and to limit head movements during the experiment to avoid the occurrence of artefacts in the electroencephalogram (EEG) recording.

Three-dimensional full-body movements were recorded using reflective markers placed on the body according to the Vicon Plug-in Gait Full Body model [24] using the Vicon Motion Systems (Oxford, UK, 100 Hz). In addition, the force plates embedded in the treadmill (Motek Medical, NL, 2000 Hz) were used for step detection. Electrical brain activity was measured using a high-density EEG with 126 Ag–AgCl electrodes (WaveGuard, ANT Neuro, The Netherlands) distributed according to the five percent electrode system [25]. EEG was recorded using a biosignal amplifier (REFA System, TMSi, The Netherlands, 2048 Hz) and MATLAB version 2018a (The MathWorks Inc. USA). Both recording systems were synchronised using a digital trigger.

### Analysis of motion capture data

Vicon Nexus 2.10.1 was used to filter (Woltring filter, Mean Square Error = 10) and pre-process the motion capture data to compute the centre of mass (CoM). Further data analyses were done using MATLAB 2019b. The ground reaction force data were filtered (zero-lag 10 Hz low-pass 4th order Butterworth filter), and the gait events (i.e., toe-off and heel strike) were determined from the force plate data using a 20N threshold. Step duration, swing duration, step length, step width, and mediolateral margin of stability (MoS) were determined for both legs. The margin of stability was defined as the minimal distance between the extrapolated centre of mass and the edge of the base of support (as determined by the lateral malleolus marker) during the stance phase [26]. Additionally, we determined the standard deviations of step width and margin of stability for each leg as measures of variability in foot placement and stability.

### EEG data processing and analysis

**Fig. 2:**
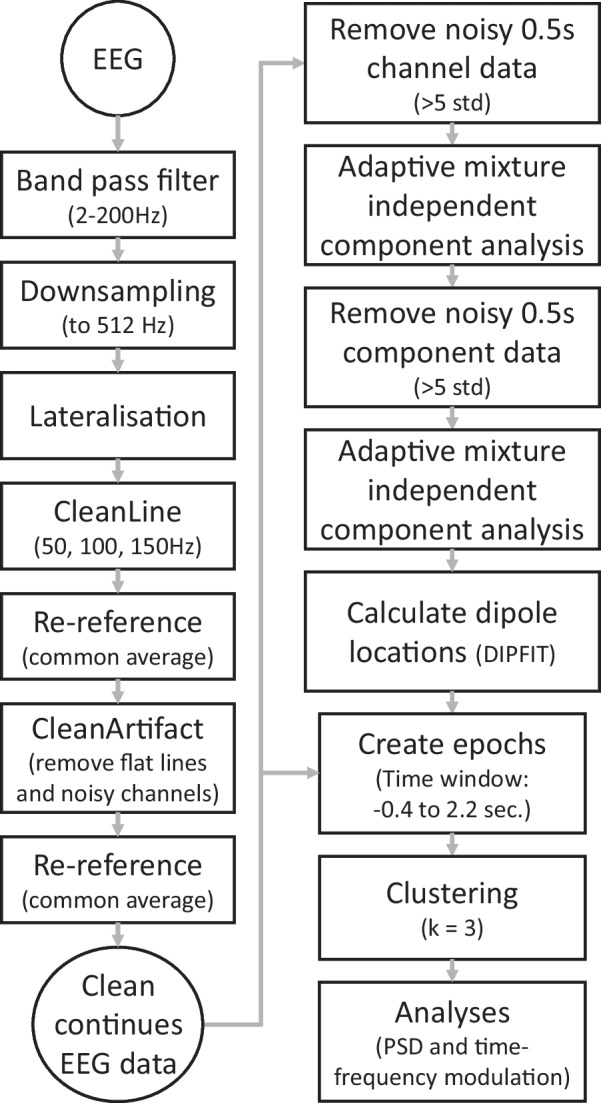
Schematic overview of the EEG data processing

A schematic overview of the EEG processing steps can be found in Fig. 2. EEG data were band-pass filtered with a zero-phase FIR filter (2–200 Hz) and down-sampled to 512 Hz. To allow clustering of lateralised brain activity across individuals with amputation on either body side, all lateralized EEG channels were switched (left to right and vice versa) of participants who had a left-sided amputation, and in the control group, whose left leg was the non-dominant leg. Hence, left-sided cortical activity in sensorimotor areas is presented corresponding to the prosthetic/non-dominant legs of our participants, whereas right-sided activity corresponds to the intact/dominant legs. Line noise harmonics (50, 100, and 150 Hz) were reduced with the CleanLine EEGLAB plugin [27, 28]. Data were re-referenced to the average reference, and the CleanArtifacts EEGLAB plugin [29] was used to remove flat lines and noisy channels if the correlation with the surrounding channels was less than 0.6. Visual inspection was performed to identify and remove noisy channels from the data. If additional channels were removed, data was re-referenced to the average reference and saved as continuous data for further analyses.

The EEG data were segmented into consecutive non-overlapping epochs of 0.5 s to remove noisy epochs with signal power higher than five standard deviations over multiple channels. The channel data was separated into components from independent brain sources using adaptive mixture independent component analysis (AMICA) [30]. The EEG component data were checked for noisy epochs. Noisy epochs were removed when the epochs had a signal power higher than five standard deviations over multiple components. This procedure was followed by a new AMICA and computing equivalent dipole locations using the DIPFIT EEGLAB plugin [31] with standardised three-shell boundary element head model and electrode positions (Montreal Neurological Institute (MNI)). Visual inspection was used to identify components resembling brain activity, with an additional criterion of residual variance of the dipole below 15%, and a dipole location within the brain volume. On average, 7 ± 6.4 (mean ± sd, range: 1–32) components were included per participant.

The retained components were projected back to the continuous data, segmented into epochs ranging from − 0.5 to 2 s (median: 174.5 gait cycles, range: 75–203), and clustered across participants using the k-means clustering algorithm (k = 7) considering the following features: mean power spectral density (PSD) (3–48 Hz), dipole locations, and associated scalp projections. Dipole locations located more than 3 times the standard deviation of distances within the cluster from the cluster centroid were considered outliers and not included in the analysis. When multiple components from one individual were present in a cluster, the components were averaged and used as a single component for the final analysis. The Yale BioImage Suite [32] was used to estimate the Brodmann area corresponding to the location of the cluster centroid.

Event-related spectral perturbation (ERSP) maps were used to compute the modulations of cortical rhythms (wavelet cycles: 1 Hz, 0.5). The ERSP maps were time-warped using both heel strike events to normalize the duration of the gait cycle to the median gait cycle duration across all participants. For each individual component, the relative power changes were computed as the average difference between the single-trial spectrogram and the average spectrogram of quiet stance (baseline). Average ERSP maps for each cluster were computed by averaging across the ERSP maps of the corresponding components for each group separately.

### Statistical analysis

Statistical analysis for the gait parameters was performed using IBM SPSS Statistics 27 (IBM B.V., the Netherlands). For the statistical analysis of the brain activity, MATLAB version 2019b (The MathWorks Inc. USA) was used. An uncorrected alpha of 0.05 was used for all statistical tests, which was deemed appropriate in light of the confirmatory nature of the comparisons between AB subjects and the TFA group at large [33, 34]. In addition, it prevented being overly conservative when exploring potential differences between the BAP and SSP subgroups.

Differences in the gait parameters between the AB and TFA group, and between BAP- and SSP-users were determined with an independent t-test or Mann–Whitney U test, depending on whether data were normally distributed. Normal distributions were tested with the Kolmogorov–Smirnov test.

To explore between-group differences in relative power modulations across the entire gait cycle, the mean PSD was compared per individual frequency (3–30 Hz) using Mann–Whitney U tests. To explore between-group differences in gait-cycle dependent modulation of brain activity, the mean ERSP map of the AB group was subtracted from the mean ERSP map of the TFA group for each cluster. A similar contrast ERSP map was made where the mean ERSP map of the BAP-users was subtracted from that of the SSP-users. A mask overlay was applied on the contrast ERSP maps to highlight average differences exceeding 1 dB. Using these contrast ERSP maps, differences in mean cortical modulation between groups will be presented descriptively for the theta (approximately 3–7 Hz), alpha (approximately 7–16 Hz), and beta band (approximately 16–30 Hz). Where an increase power in the theta band is related to additional performance monitoring and a decrease in the alpha and beta band is associated with increased motor planning and motor control.

## Results

### Participants

Demographics for the AB and TFA groups and the BAP- and SSP- users are shown in Table 1. Between the AB and TFA groups, no significant differences in demographics were found. Within the TFA group, the SSP-users had a significantly higher weight of the prosthesis (t(10,4) = − 2.540, p = 0.028) and longer residual limb length (t(18) = − 3.987, p < 0.001) compared to the BAP-users.

**Table 1:** Mean ± standard deviation of participants’ demographics for AB and TFA, and BAP- and SSP-users

	AB (n = 18)	TFA (n = 20)	BAP (n = 10)	SSP (n = 10)
Age (years)	55 ± 11	57 ± 13	59 ± 15	56 ± 13
Sex (m/f)	10/8	11/9	6/4	5/5
Length (cm)	177 ± 8	175 ± 10	175 ± 10	176 ± 10
Weight (kg)	78 ± 15	78 ± 16	77 ± 13	80 ± 20
Cause of amputation			5 tr, 4 ca, 1 con	8 tr, 1 ca, 1 inf
Years since amputation			25 ± 17	27 ± 14
Years since the OI operation			5 ± 2	
Weight of prosthesis (kg)			**3.5 ± 0.3**	**4.3 ± 1.0**
Residual limb length (cm)			**15.3 ± 3.8**	**21.9 ± 3.5**
Prosthetic knees			6 × C-Leg, Rheo XC, Freedom Flie, VGK, 3R80	5 × Genium, 2 × C-Leg, 2 × VGK, 3R106 Pro

Bold fonts indicate p-values below 0.05; AB: able-bodied persons; TFA: persons with a transfemoral amputation; BAP: bone-anchored prosthesis group; SSP: socket-suspended prosthesis group; m: male; f:female; cm: centimetre; kg: kilogram; OI: osseointegrated implant tr: trauma; ca: cancer; inf: infection; con: congenital

### Gait parameters

The gait parameters show a significantly lower gait speed in the TFA group compared to AB subjects (t(36) = 3.155, p = 0.003, Table 2). In addition, compared to AB subjects, the TFA group showed significantly longer step (U = 47, p < 0.001) and swing phase duration (U = 39, p < 0.001) for the prosthetic/non-dominant leg, smaller step length for both the prosthetic/non-dominant (t(36) = 2.227, p = 0.032) and intact/dominant leg (U = 84, p = 0.005), and larger step width (t(36) = − 2.916, p = 0.006). Regarding gait variability, the TFA group demonstrated a larger standard deviation of the step width (t(36) = − 2.364, p = 0.024) than the AB group. No significant between-group differences were observed in the margins of stability outcomes. Within the TFA group, no significant differences in the gait parameters were found between the BAP- and SSP-users.

**Table 2:** Mean ± standard deviation of gait measures for AB and TFA group, and BAP- and SSP-users

	AB (n = 18)	TFA (n = 20)	BAP (n = 10)	SSP (n = 10)
Walking speed (km/h)	**3.8 ± 0.7**	**3.1 ± 0.7**	3.2 ± 0.8	3.0 ± 0.6
Step duration (sec.)				
Prosthetic leg	**0.58 ± 0.07**	**0.65 ± 0.05**^**A**^	0.65 ± 0.04	0.66 ± 0.05^A^
Intact leg	0.57 ± 0.07	0.58 ± 0.04^A^	0.56 ± 0.04	0.59 ± 0.04
Swing phase duration (sec.)				
Prosthetic leg	**0.42 ± 0.05**	**0.46 ± 0.03**^**A**^	0.46 ± 0.03	0.47 ± 0.03
Intact leg	0.40 ± 0.05	0.38 ± 0.03	0.38 ± 0.03	0.38 ± 0.03
Step length (cm)				
Prosthetic leg	**55.5 ± 7.6**	**48.3 ± 11.7**	48.8 ± 11.7	47.7 ± 12.6
Intact leg	**56.4 ± 9.1**	**48.0 ± 8.4**^**A**^	48.5 ± 9.5	47.5 ± 7.7
Step width (cm)	**11.9 ± 3.0**	**14.7 ± 2.8**	14.7 ± 2.8	14.7 ± 2.9
Step width SD (cm)	**1.9 ± 0.4**	**2.3 ± 0.5**	2.3 ± 0.5	2.2 ± 0.5
MoS (cm)				
Prosthetic leg	7.2 ± 2.1	8.0 ± 1.8	8.0 ± 1.6	8.0 ± 2.0
Intact leg	6.9 ± 1.3	7.9 ± 1.7^A^	8.5 ± 1.7	7.4 ± 1.6
MoS SD (cm)				
Prosthetic leg	1.2 ± 0.7	1.5 ± 0.9^A^	1.4 ± 0.8	1.6 ± 0.9
Intact leg	1.1 ± 0.5	1.7 ± 1.1^A^	1.7 ± 1.1	1.6 ± 1.1

Statistics were done using an independent t-test unless otherwise indicated; ^A^ Statistically tested using Mann Whitney U test; Bold indicates p-value below 0.05; For the AB group, intact leg indicated the dominant leg and the prosthetic leg indicated the non-dominant leg. AB: able-bodied persons; TFA: persons with a transfemoral amputation; BAP: bone-anchored prosthesis group; SSP: socket-suspended prosthesis group; km/h: kilometer per hour; sec.: seconds; cm: centimetre; SD: standard deviation; MoS: Margin of stability

### Clusters of independent components

**Fig. 3:**
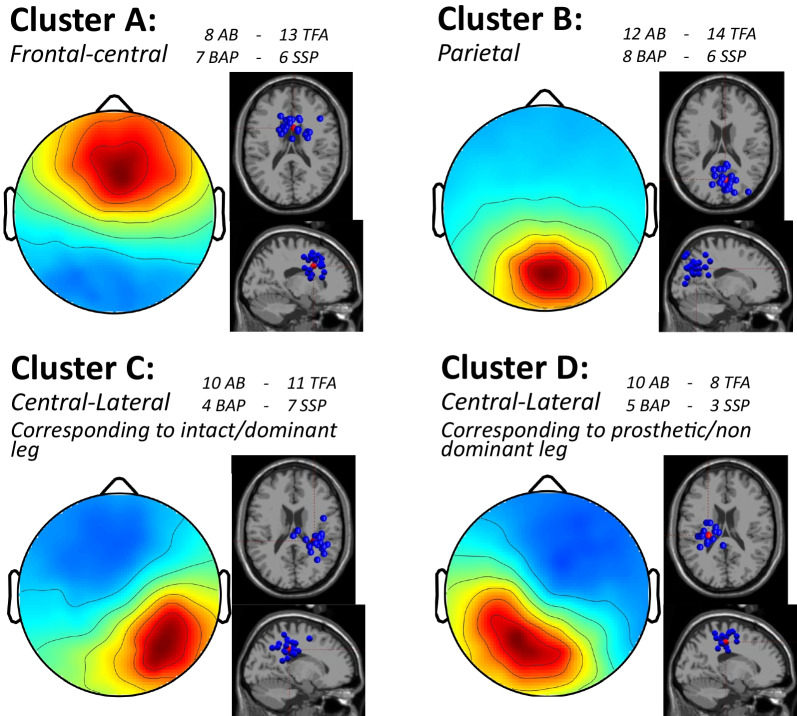
Scalp projections and dipole locations of the four clusters. The blue dipoles represent the location of individual independent components, and the red dipoles represent the cluster centroid. For each cluster the number of included participants/independent components are presented. AB: able-bodied persons; TFA: persons with a transfemoral amputation; BAP: bone-anchored prosthesis group; SSP: socket-suspended prosthesis group

We identified four clusters, each containing independent components from more than half of the number of participants. One subject in the AB group was excluded from the EEG analysis after visual inspection of the individual PSDs indicated this subject was an outlier compared to remainder of the AB group. Figure 3 displays the corresponding scalp projections and dipole locations. The MNI coordinates of the mean cluster centroids locations are [3, 11, 30] for cluster A (Brodmann area 24), [5,− 62,30] for cluster B (Brodmann area 7), [29, − 29,34] for cluster C (nearest Brodmann area 2), and [− 25, − 22,44] for cluster D (nearest Brodmann area 4). Therefore, the clusters were identified as frontal-central (A), parietal (B), central-lateral right (corresponding to the intact/dominant leg, C), and central-lateral left cortex (corresponding to prosthetic/non-dominant leg, D), respectively.

### Mean PSDs and modulations of cortical rhythms within the gait cycle

**Fig. 4:**
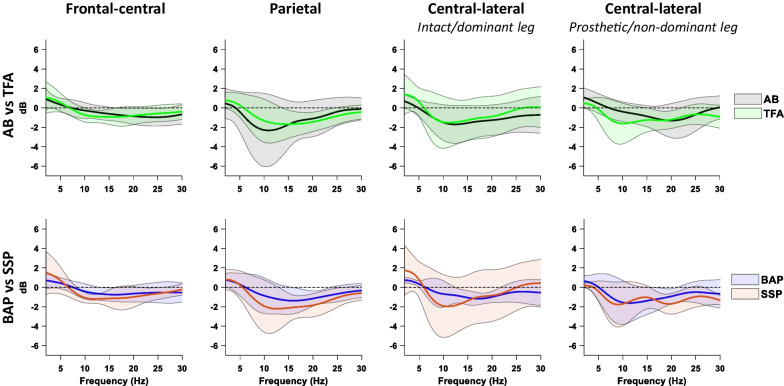
mean power spectral density of walking with a quiet stance baseline subtracted of each cluster and group. The shaded area represents the standard deviation. AB: able-bodied persons; TFA: persons with a transfemoral amputation; BAP: bone-anchored prosthesis group; SSP: socket-suspended prosthesis group

Figure 4 shows the mean PSDs over the gait cycle after subtraction of quiet stance as baseline. In all clusters for all groups, the alpha and beta band mean power was decreased during gait compared to quiet stance. Further, the AB group displays a large variability in the parietal cluster across all frequency bands. Except for the central-lateral cluster corresponding to the prosthetic/non-dominant leg, the mean theta power (approximately 2–7 Hz) seems, on average, higher in the TFA group than in the AB group. However, no statistically significant differences in the mean power over the gait cycle were found between the TFA and AB group, or between BAP- and SSP-users.

**Fig. 5:**
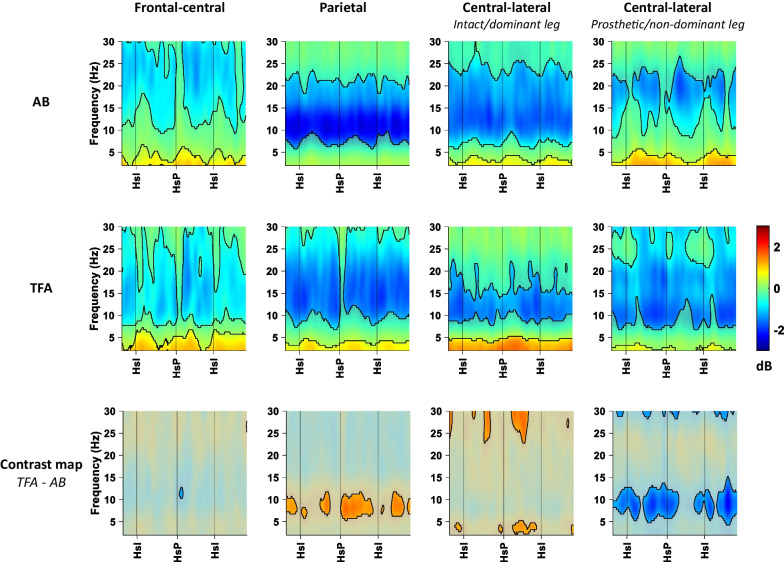
ERSP maps and contrast maps for each cluster of AB and TFA. Time–frequency maps show the decrease (blue) and increase (red) in mean power for each condition. In the ERSP maps of AB and TFA, the non-significant differences from quiet stance are partially masked with a white overlay. The contrast maps display the difference in power between TFA and AB, the differences between − 1 and 1 dB are masked with a gray overlay. AB: able-bodied persons; TFA: persons with a transfemoral amputation; HsI: heel strike with intact/dominant leg; HsP: heel strike with prosthetic/non-dominant leg

Mean ERSP maps with a baseline of quiet stance and the contrast maps for the AB and TFA group are shown in Fig. 5. In both the AB and TFA groups, the ERSP map of every cluster shows decrements in alpha and beta power and a general increment in theta power compared to the quiet standing baseline.

- The contrast map of the frontal-central cluster indicates stronger power decrease in the alpha band for only a very brief moment after heel strike of the prosthetic/non-dominant leg for TFA compared to the AB group.
- The contrast map of the parietal cluster mainly indicates a lower alpha power decrease in the TFA group compared to the AB group during the entire gait cycle.
- For the central-lateral cluster corresponding to the intact/dominant limb, the contrast map indicates a greater power increase in the theta band just before heel strike of either legs and during intact/dominant swing phase for the TFA group compared to AB.
- In the central-lateral cluster corresponding to the prosthetic/non-dominant leg, the TFA group seems to have a decrease in power of the alpha band during heel strike with either leg and during the swing phase of the prosthetic/non-dominant leg in comparison to the AB group.

**Fig. 6:**
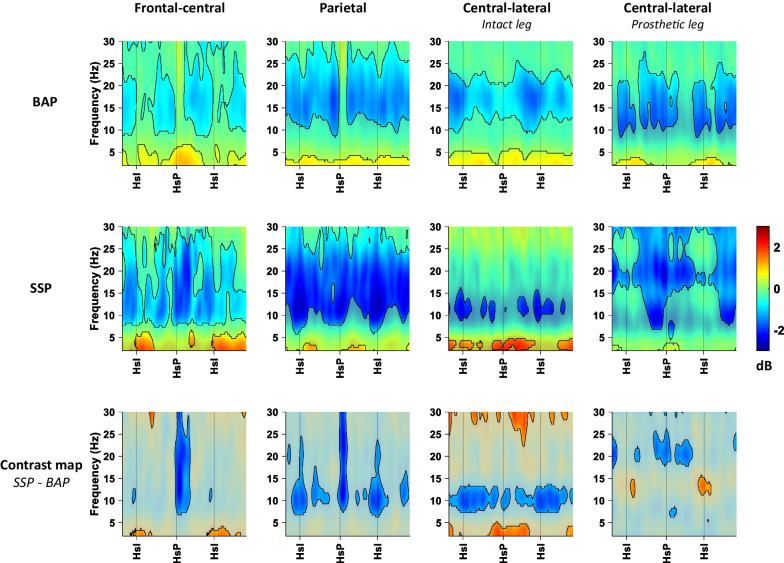
ERSP maps and contract maps for each cluster of BAP and SSP. Time–frequency maps show the decrease (blue) and increase (red) in mean power for each condition. In the ERSP maps of BAP and SSP, the non-significant differences from quiet stance are partially masked with a white overlay. The contrast maps display the difference in power between BAP and SSP, the differences between − 1 and 1 dB are masked with a gray overlay. BAP: bone-anchored prosthesis group; SSP: socket-suspended prosthesis group; HsI: heel strike with the intact leg; HsP: heel strike with a prosthetic leg

For the BAP- and SSP-users, the mean ERSP maps (with a baseline of quiet stance) and the contrast maps are shown in Fig. 6. In both BAP- and SSP-users, the ERSP maps of all clusters show significant increments in theta power, and decrements in alpha and beta power in a gait-cycle dependent pattern relative to quiet stance.

- In the frontal-central cluster, the BAP- and SSP-users show increments in theta power mainly around heel strike. The contrast map of the frontal-central cluster shows a stronger power decrease in the alpha and beta bands after heel strike of the prosthetic leg in the SSP-users compared to the BAP-users.
- In the parietal cluster, the contrast map indicates stronger decrements in alpha and beta power in the SSP-users compared to the BAP-users, which are observed most prominently around the heel strike of either leg.
- The contrast map of the central-lateral cluster corresponding to the intact limb indicates a greater power increase of the (low) theta band for the SSP-users compared to the BAP-users, mainly just before and during the initial stance phase of the prosthetic leg. Furthermore, power decrements in the alpha band seem to be greater during the entire gait cycle in the SSP- compared to the BAP-users, most strongly following heel strike of the intact limb.
- For the central-lateral cluster corresponding to the prosthetic leg, the contrast map indicates a brief episode of weaker power decrease in the alpha band for SSP-users around heel strike of the intact leg compared to BAP-users. In the beta band, SSP-users show a greater power decrease just before heel strike and during the initial *s*tance phase of the prosthetic leg compared to BAP-users.

## Discussion

The current study aimed to determine differences in gait pattern and cortical dynamics during gait between persons with transfemoral amputation (TFA) and able-bodied persons (AB), as well as between persons using a socket-suspended (SSP) and bone-anchored prosthesis (BAP). Differences in spatiotemporal and stability-related gait parameters were found between persons with TFA and AB, yet no significant differences were found between BAP- and SSP-users. Four relevant cortical clusters were identified in frontal, central, and parietal areas. Time–frequency analyses yielded preliminary insight into differential power modulations of the theta, alpha, and beta bands between TFA and AB groups, and between BAP- and SSP-users.

The current findings that persons with TFA walked slower with longer prosthetic step and swing phase duration, smaller step lengths and step width, and greater variability in mediolateral foot placement compared with AB persons are in line with previous research [4, 35, 36]. However, even though previous studies [35, 37] demonstrated greater CoM and trunk excursions in persons with TFA compared to AB during walking, this did not result in differences in margin of stability (MoS). This may indicate that, similar to other clinical populations [38–40], persons with TFA appeared to lower their walking speed and increase their step width as a compensatory mechanism for maintaining margins of stability comparable to those of AB subjects. In addition, greater variability in mediolateral foot placement was found in the absence of greater variability in MoS. These findings may imply that persons with TFA use the additional variability in step width to effectively compensate for the more variable centre of mass excursions [37, 41, 42], which is consistent with the suggested key role of foot placement for controlling CoM dynamics during gait [43, 44].

No differences between BAP- and SSP-users were found in any of the gait parameters in the present study, indicating that the prosthetic suspension has limited effect on the gait pattern or gait stability when level walking with a transfemoral prosthesis. This is consistent with previous research between BAP- and SSP-users [45], and regarding socket design [46], which also demonstrated no to only minor changes in step length symmetry and step width. These results therefor indicate that the benefits seen with provision of a BAP in persons with socket-related problems [47–49] cannot be generalized to highly-active socket-suspended users. However, it could be argued that level ground walking is not challenging enough to evaluate the potential influence of prosthetic suspension on the gait pattern or gait stability. Indeed, MoS and variability in MoS have been shown to increase during perturbed gait in persons using a leg prosthesis [50]. To better understand the impact of the prosthetic suspension on gait pattern and stability, also under more complex daily-life walking conditions, further research using challenging tasks and environments is required.

Exploratory analyses on cortical patterns revealed stronger increase of theta band power in the TFA group compared to the AB group in the central-lateral cluster corresponding to the intact limb. Increased theta power in these cortical areas has previously been linked to (postural) error detection (i.e. performance monitoring) [13–16]. The present observations of differential theta power modulation may suggest that compared to AB persons, the TFA group showed greater engagement of these cortical areas in monitoring (dis)agreement between the internal model and the actual execution of the movement. This interpretation would be in line with the greater step width and MoS variability in the TFA group, as discussed above.

While no differences were observed in gait parameters between the two TFA groups, the ERSP maps suggest that (postural) performance monitoring during the gait cycle may differ between BAP- and SSP-users. During the prosthetic stance phase, the SSP-users seem to demonstrate more increased central-lateral theta power (corresponding to the intact limb) compared to BAP-users. The observation might be related to the additional performance monitoring needed with the intact limb to compensate for the less stablenon-rigid connection of the socket suspension in comparison to the BAP-users with their fixed connection of the osseointegrated implant. With the less stable connection of a socket suspension, the movement and placement of the intact limb may be more important to maintain stability during gait. This may require additional performance monitoring of the intact limb, potentially resulting in similar gait parameters between SSP- and BAP-users.

In both central-lateral clusters, global power decrements in the alpha band were observed. In the cluster corresponding to the prosthetic/non-dominant leg, this power decrement appeared to be stronger in persons using a TFA compared to AB subjects (Fig. 5, bottom-right). As a decrease in alpha power is suggested to be related to motor planning and execution [17–20], this observation may point at persons with a TFA exerting greater cortical control of prosthetic-leg movement planning and execution during gait compared to the non-dominant leg of AB persons. Within the TFA group, this seemed to apply rather similarly to both groups, except around the intact-leg heel strike (Fig. 6, bottom-right). However, in the central-lateral cluster corresponding to the intact leg, we observed more prominent alpha power decrements in the SSP- than in the BAP-users, which emerged more strongly during the stance phase of the intact leg (Fig. 6, bottom second from the right). This observation may suggest that the SSP-users exert greater intact-leg movement planning and execution in this phase of the gait cycle. In line with our reasoning regarding theta power differences, the alpha power differences may also be related to the intact limb presumably compensating for the less stable non-rigid connection of the socket suspension. Intact-leg movements may therefore require more precise planning and execution to maintain stability with the inherently less stable stance phase of the prosthetic leg in SSP users.

In the frontal-central cluster, no mean differences between persons with TFA and AB subjects were observed that exceeded 1dB, except for a brief moment of stronger alpha power decrease in the TFA group right after prosthetic heel strike (Fig. 5, bottom-left), which may point at increased motor planning [21, 22]. This increased motor planning might be attributed to the additional stabilisation exerted by the hip muscles [4], particularly during weight acceptance of the prosthetic leg. One might have expected to also see a decrease in beta band power in the TFA group, because decreased beta power in this cluster has been linked to increased cognitive control [21, 22]. The lack of between group differences may be related to the normalized of power modulations to a quiet standing baseline. As previous studies have found differential effects of dual-tasking on quiet standing postural control in persons with a lower-limb amputation [10], the baseline condition may already have been more cognitively demanding for our TFA group compared to the AB persons.

In the frontal-central and parietal cluster, a stronger alpha and beta power decrease is observed in the SSP-users compared to the BAP users. In the frontal-central cluster, this occurred mainly around prosthetic heel strike, and in the parietal cluster around heel strike of either leg. Previous studies have reported that a decrease in alpha and beta power in the frontal central and parietal cortex is linked to increased motor planning and motor control [21, 51, 52]. It may be speculated that the relative less stableconnection of the socket may necessitate such additional motor planning and motor control in SSP users, mainly around heel strike of the prosthetic leg.

There are several study limitations and considerations that should be noted. First, residual limb length and prosthetic weight were significantly different between the BAP and SSP group. These differences did not translate to between-group difference in spatiotemporal gait parameters, which is in line with previous findings[53, 54]. Yet, as research on cortical activation patterns in people with TFA is still in its infancy, it is unknown whether differences in residual limb length and prosthetic weight might potentially be relevant as mediators. In addition, a limitation for interpreting the cortical activity analysis is the sample size, which particularly concerns the SSP and BAP groups. Some of the clusters of cortical activity consequently contained components of only a part of the full sample. For this reason, we refrained from formal statistical testing of between-group differences of the ERSP maps and, instead, chose to present average differences exceeding an arbitrary 1dB threshold descriptively. It goes without saying that these preliminary findings from our exploratory study have to be interpreted with caution. Another limitation is that all lateralized EEG channels were switched (left to right and vice versa) of participants who had a left-sided amputation, to allow clustering of lateralized brain activity according to the side of amputation. This may have obscured ‘normal’ lateralized activity (i.e. unrelated to walking with a prosthesis). Yet, there is currently little evidence for such ‘normal’ lateralized brain activity in other cortical areas during gait. Furthermore, the use of a template head model for source localization may reduce the localization accuracy of individual components. However, the group-level analysis (indicated by the cluster centroid) may offer a more precise estimate of the cortical source location. As the ERSP maps show similar modulations of cortical activity to those previously reported for these cortical regions during gait, we believe that interpretations on between-group differences can be made against this background of existing knowledge of cortical gait control.

Taking these points into consideration, some interesting cortical patterns appear to emerge, which may provide directions for future research. Walking tasks that challenge gait stability, like mediolateral perturbations, could be used to further investigate potential differences in gait and underlying cortical patterns related to the prosthetic suspension.

## Conclusions

Contrary to our assumption, the current study found no differences in the gait parameters between the BAP- and SSP-users, whereas the cortical patterns might suggest differences in the underlying neural control processes. In addition, changes in gait pattern and gait stability between persons using a transfemoral prosthesis and able-bodied persons were confirmed, and preliminary insights were gained of gait-cycle dependent modulations in cortical dynamics pointing at some emerging differences in the frontal-central, parietal, and central-lateral brain areas that appear (partly) consistent with prosthetic users more actively monitoring and controlling their gait.

## Data Availability

The datasets used and/or analysed during the current study are available from the corresponding author on reasonable request.

## Abbreviations

AB: Able-bodied person
AMICA: Adaptive mixture independent component analysis
BAP: Bone-anchored prosthesis
CoM: Centre of mass
EEG: Electroencephalogram
ERSP: Event-related spectral perturbation
MNI: Montreal Neurological Institute
MoS: Margin of Stability
PSD: Power spectral density
TFA: Transfemoral amputation
SSP: Socket-suspended prosthesis
